# BRCC3 Promotes Tumorigenesis of Bladder Cancer by Activating the NF-κB Signaling Pathway Through Targeting TRAF2

**DOI:** 10.3389/fcell.2021.720349

**Published:** 2021-09-16

**Authors:** Huangheng Tao, Yixiang Liao, Youji Yan, Zhiwen He, Jiajie Zhou, Xinghuan Wang, Jianping Peng, Shangze Li, Tao Liu

**Affiliations:** ^1^Department of Urology, Zhongnan Hospital of Wuhan University, Wuhan, China; ^2^Medical Science Research Center, Zhongnan Hospital of Wuhan University, Wuhan, China; ^3^State Key Laboratory Breeding Base of Basic Science of Stomatology (Hubei-MOST) and Key Laboratory for Oral Biomedicine of Ministry of Education (KLOBM), School and Hospital of Stomatology, Wuhan University, Wuhan, China; ^4^Jingzhou Hospital, Yangtze University, Jingzhou, China; ^5^The Second Clinical Medical College, Yangtze University, Jingzhou, China; ^6^Hubei Key Laboratory of Cell Homeostasis, College of Life Sciences, Wuhan University, Wuhan, China; ^7^School of Medicine, Chongqing University, Chongqing, China

**Keywords:** BRCC3, TRAF2, NF-kB, tumorigenesis, bladder cancer

## Abstract

NF-κB signaling is very important in cancers. However, the role of BRCC3-associated NF-κB signaling activation in bladder cancer remains to be characterized. Western blotting and IHC of tissue microarray were used to confirm the abnormal expression of BRCC3 in bladder cancer. Growth curve, colony formation, soft agar assay and Xenograft model were performed to identify the role of BRCC3 over-expression or knock-out in bladder cancer. Further, RNA-Seq and luciferase reporter assays were used to identify the down-stream signaling pathway. Finally, co-immunoprecipitation and fluorescence confocal assay were performed to verify the precise target of BRCC3. Here, we found that high expression of BRCC3 promoted tumorigenesis through targeting the TRAF2 protein. BRCC3 expression is up-regulated in bladder cancer patients which indicates a negative prognosis. By *in vitro* and *in vivo* assays, we found genetic BRCC3 ablation markedly blocks proliferation, viability and migration of bladder cancer cells. Mechanistically, RNA-Seq analysis shows that NF-κB signaling is down-regulated in BRCC3-deficient cells. BRCC3 binds to and synergizes with TRAF2 to activate NF-κB signaling. Our results indicate that high BRCC3 expression activates NF-κB signaling by targeting TRAF2 for activation, which in turn facilitates tumorigenesis in bladder cancer. This finding points to BRCC3 as a potential target in bladder cancer patients.

## Introduction

Bladder cancer (BCa) is one of the most common tumors in the urinary system. There are approximately 78,100 new cases and 32,100 bladder cancer-related deaths in China annually (Cumberbatch et al., 2018). The bladder cancer related incidence and mortality rates in China have been rising rapidly. At present, diagnosing bladder cancer mainly depends on urine exfoliative cytology, cystoscopy and live examination, of which the former has very low sensitivity and the latter is an intrusive operation increasing the suffering of the patients (Roos et al., 2008; He et al., 2018; Choudhury and Hoskin, 2019). The main method of clinical treatment for bladder cancer is surgical treatment. Once the opportunity for surgical intervention is lost or the disease recurs, treatment can rely on only traditional radiotherapy and chemotherapy, and there is no molecular targeted drug suitable for bladder cancer in the clinic. The main reason for this clinical approach is the lack of accurate and reliable molecular therapeutic markers for bladder cancer. Although triple therapy with surgery/radiotherapy/chemotherapy has recently been explored to treat advanced bladder cancer, the efficacy is still not good enough (Dudley et al., 2018; Efstathiou et al., 2019). Therefore, to explore the mechanism of bladder cancer progression, and identify specific diagnostic molecules and potential therapeutic targets for bladder cancer are quite urgently needed.

The NF-κB signaling pathway plays important roles in various kinds of chronic diseases, including cancer, inflammation and so on (Kunnumakkara et al., 2018). The aberrant activation of NF-κB signaling pathway has been noted in various human cancers (Rayet and Gelinas, 1999; Al-Halabi et al., 2011). Hence, targeting the NF-κB pathways have high potential for preventing cancers. However, most drugs targeting the NF-κB pathway have adverse side effects. Therefore, it is urgent to find a novel protein targeting NF-κB pathways that plays a crucial role in bladder cancer (Kunnumakkara et al., 2018).

BRCC3 is a member of deubiquitinase family, which can specifically cleave lysine 63-linked ubiquitin polymers (K63-Ub). The zinc-dependent JAB1/MPN/MOV4 metalloprotease domain is critical for the deubiquitylation activity of BRCC3 (Huang et al., 2015). In the DNA damage response, BRCC3 specifically cuts K63-Ub from histones H2A and H2AX. DNA double-strand breaks (DSBs) can result in genotoxic stress and promote tumorigenesis (Sun et al., 2016). Consistent with this conclusion, aberrant expression of BRCC3 has been associated with decreased sensitivity to ionizing radiation breast carcinomas and defects in the G2/M checkpoint in nasopharyngeal carcinomas (Dong et al., 2003; Tu et al., 2015). In cervical cancer, knocking down BRCC3 expression inhibits cell viability, invasion and migration via inhibition of epithelial-mesenchymal transition (EMT) progression and the expression levels of Snail family (Zhang and Zhou, 2018). Apart from its role in the DSB-associated carcinogenesis, BRCC3 regulates the type I interferon signaling pathway during antiviral responses and inflammasome activity by deubiquitinating NLRP3 (Py et al., 2013; Hu et al., 2019). Although BRCC3 has emerged as an oncogene in various tumors, the role of BRCC3 in bladder cancer is still obscure.

In this study, we reported BRCC3 as a novel oncogene in bladder cancer. We identified BRCC3 bound to TRAF2 to activate NF-κB signaling. Meanwhile, we found BRCC3 was aberrantly expressed in BCa patients with an unfavorable prognosis. BRCC3 over-expression promoted tumorigenesis and was not associated with the enzymatic activity of BRCC3. In contrast, knocking out of BRCC3 attenuated the tumorigenesis *in vitro* and *in vivo*. Our findings suggested BRCC3 as a potential target in BCa patients.

## Materials and Methods

### Cell Culture

Immortalized human bladder epithelial SV-HUC1 cell; human bladder cancer cells lines including the EJ, T24, 5637, UMUC3, SW780, RT4, and J82 cell lines; and HEK293T cell line were purchased from the Stem Cell Bank, CASS, China. SV-HUC1, EJ, T24, and 5637 cells were cultured in RPMI 1640 medium containing 10% fetal bovine serum (FBS; Gibco, China), 100 U/ml penicillin-G sodium and 100 mg/ml streptomycin sulfate at 37°C under an atmosphere of 95% air and 5% CO2. UMUC3, SW780, RT4, J82, and HEK293T cells were cultured in Dulbecco’s modified Eagle’s medium (DMEM) (Gibco) supplemented with 10% FBS, 100 U/ml penicillin-G sodium and 100 mg/ml streptomycin sulfate at 37°C in 5% CO_2_.

### Reagents and Antibodies

Proteasome inhibitor MG132 (Sigma, United States); the protein translation inhibitor cycloheximide (CHX, Sigma); and low melting point agarose (Solarbio Life Sciences, Spain) were purchased. The cDNA Reverse Transcription Kit was purchased from Thermo Fisher Scientific (Thermo Fisher Scientific, United States). Antibodies against BRCC3 (1:1,000, Cat. #18215), TNFα (1:100, Cat. #8184), and Ki67 (1:200, Cat. #9449) were purchased from Cell Signaling Technologies (Danvers, MA, United States). An anti-β-actin antibody (1:1,000, Cat. #AC026) was purchased from ABclonal (ABclonal, United States). Antibodies against FLAG (1:1,000, Cat. #M185-3L) and HA (1:1,000, Cat. # M180-3) were purchased from MBL (MBL Beijing Biotech Co., Ltd.). An anti-TRAF2 antibody (1:1,000, Cat. #ab126758) was obtained from Abcam (Abcam, United Kingdom).

### Plasmids and sgRNA

A plasmid expressing wild type BRCC3 and the deubiquitinating enzyme-null mutant BRCC3 (which was built by a site-directed mutation of H122Q) was generated using PCR and cloned into pHAGE/puro/3 × Flag. Plasmids expressing HA-TRAF2, HA-TNFR, HA-TAK1, HA-TAB1, HA-IKKβ, or HA-P65 were generated with pCDNA5/FRT/TO-3 × HA, respectively. pRL-TK and pGL3-NF-κB-luc were purchased from Addgene (Cambridge, MA, United States). For CRISPR-Cas9 gene editing, small guide RNAs (sgRNAs) of BRCC3 were cloned into lenti-v2 (Addgene, Cat. #92062). The sgRNA sequences: sgRNA-F, 5′-caccGAAGTAATGGGGCTGTGCAT-3′; sgRNA-R, 5′-aaacATGCACAGCCCCATTACTTC-3′.

### Genetic Knock-Out of BRCC3 in Human Bladder T24 Cells

The genetic knockout of BRCC3 in T24 cells was performed using a CRISPR-Cas9 system. Briefly, we designed BRCC3-sepcific sgRNAs^1^ which was cloned into lenti-v2. HEK293T cells were transfected with two packaging vectors and the recombinant lenti-v2 vector containing the BRCC3-sepcific sgRNA sequences. After 48 h, the supernatant containing the lentivirus was harvested. 1 × 10^5^ T24 cells were incubated with the lentivirus for 48 h and then exposed to 600 ng/ml puromycin for another 5 days. Next, the cells were put in five 96-well plates by limiting dilution method. After 14 days of growth, the single clones were screened using immunoblotting analysis with an anti-BRCC3 antibody, and the positive clones were amplified.

### Transient Transfections and Lentivirus-Mediated Stable Overexpression

To produce lentivirus, 293T cells were incubated with MAX transfection reagent, corresponding expression plasmids together with the packaging vectors pMD2.G (Addgene, Cat. #12259) and psPAX2 (Addgene, Cat. #12260). The transfection complex was mixed in DMEM (Gibco) without FBS, and finally dripped to cells. And the medium which contains virus was gain 72 h later. For stable transfection with pHAGE-3 × FLAG-BRCC3, 600 ng/ml puromycin was used for 5 days to kill the non-transfected cells. The efficiencies of transfection were determined by western blot.

### Cell Proliferation Assays

We used Electric Cell-substrate Impedance Sensing (ECIS) method to perform cell proliferation assays. An ECIS system was obtained from Applied BioPhysics, Inc. The ECIS culture-ware used was 96W10idf. Briefly, the protocol was as follows: The cell suspension was prepared first, and then counted. The cell suspension was diluted to 4 × 10^3^ cells in 200 μl, and added to a 96-well electrode plate. Then, the signal from the cells in the electrode plate was conventionally monitored for 48–72 h. The data were analyzed by the specific software matched to the ECIS system. The experiments were performed in triplicate.

### Colony Formation and Soft Agar Assay

For the colony formation assay, UMUC3 cells (4 × 10^2^ cells) or T24 cells(1 × 10^3^ cells)were cultured in a 6-well plate. Eight days later, the cell colonies were fixed and stained with 0.3% crystal violet in ethanol, counted and photographed. For the soft agar assay, low melting point agarose and 2 ml of 0.7% lower agar-RPMI 1640 medium were plated into 6-well plates. Then, UMUC3 cells (3 × 10^4^) or T24 cells (5 × 10^4^ cells) was mixed with agar-RPMI 1640 medium, which were cultured for more than 14 days. Finally, the numbers of cell clones were counted and recorded (the cell colony including more than 10 cells was counted as one colony).

### Luciferase Reporter Assays

For luciferase assays, a dual-luciferase kit [Promega, Cat. #E1980, Promega (Beijing) Biotech Co., Ltd.] was used. 48 h after cells were transfected with pGL3-NF-κB-luc (containing a repetitive NF-κB sequence), pRL-TK and the indicated levels of the expression constructs, the reporter assays were performed according to the standard protocol.

### Real-Time PCR

Total RNA was isolated using TRIzol Reagent (Invitrogen). The RevertAid Fist Strand cDNA Synthesis Kit (Thermo Fisher Scientific) was used for the RT analysis, and 2 μg of RNA was reverse transcribed into cDNA. Real-time quantitative PCR was performed by adding 2 μl of RT reaction mixture to a final volume of 20 μl and analyzing the reaction mixture with an ABI PRISM 7500 system (Applied Biosystems, Forster City, Calif) by using the FastStart Universal SYBR Green Master protocol (ROCHE, 04913850001). Primer sequences and annealing temperatures are summarized in Supplementary Table 1. Values were normalized to GAPDH amplification.

### Immunoblotting Analysis

Cells were washed by ice-cold phosphate-buffered saline (PBS) for three times and lysed in 1% Triton lysis buffer on ice. A BCA kit (Thermo Fisher Scientific) was then used to test the protein concentrations. 50 μg total protein (was separated by 10–15% SDS-PAGE electrophoresis (Promoton Biotechnology, shanghai, China) and transferred onto PVDF membranes (Millipore, cat# IPVH00010). After electrophoresis, PVDF membranes with protein on it were incubated with TBST (containing 5% non-fat dry milk) for 60 min at room temperature, and then incubated with specific primary antibodies overnight at 4°C followed by incubation with HRP-conjugated secondary antibodies at 37°C for 2 h at room temperature. The membranes were developed with the WesternBright ECL HRP substrate (Advansta).

### Co-immunoprecipitation

NP-40 lysis buffer was used to lysed cells in immunoprecipitation assays. The indicated antibody and protein G-agarose beads (Roche) were added into the cell lysates at 4°C overnight. Then, the beads were washed three times with 500 μl of wash buffer containing 300 mM NaCl at 4°C. The precipitates were analyzed by standard western blot.

### Fluorescence Confocal Assays

T24 cells were seeded in 12-mm cover-slips, and washed with PBS. After that, cells were fixed with 4% PFA for 15 min. The cells were then treated with a 0.1% Triton X-100 solution and blocked in normal goat serum for 30 min at room temperature. The fixed cells were incubated with the indicated antibodies at the proper dilution for 2 h at room temperature, washed three times with PBS, and incubated with secondary antibodies for 1 h. Nuclei were visualized by incubating with DAPI (2 μg/ml) for 10 min at room temperature, and slides were analyzed using a confocal microscope system (Nikon C2^+^ Confocal Microscope, Japan).

### Xenograft Assays

Male BALB/c-null mice (4-weeks old) were purchased from Beijing Vital River Laboratory Animal Technology Co., Ltd. (Beijing, China), and maintained in the laboratory animal facility of Zhongnan Hospital of Wuhan University. One week later for adaptive feeding, mice were subcutaneous injected with 4 × 10^6^ wide-type T24 cells or BRCC3^–/–^ T24 cells (*n* = 6). Five weeks later, the mice were sacrificed under effectively anesthetic by 2% pentobarbital sodium (30 mg/kg) and the tumors were removed and weighed. In addition, tumor volume was measured every 3 days.

### Immunohistochemical (IHC) Analysis

Two tissue microarrays (Alenabio, cat. #BL2081c, and cat. #T124a, Alenabio Co., Ltd) including 188 bladder cancer tissue specimens, 12 corresponding adjacent tissues specimens and 16 normal bladder tissue specimens were used. Briefly, the paraffin-embedded sections were first deparaffinized. And then citrate buffer (pH 6.0) was used for antigen retrieval, and 0.3% H_2_O_2_ was used to block the endogenous peroxidase activity. The indicated primary antibody and secondary antibody were added to the sections. Nuclei were labeled with DAB. The histoscore value of BRCC3 in the paraffin-embedded sections was analyzed by fluorescence microscopy.

### Statistical Analysis

SPSS version 13.0 (University of Nevada, Las Vegas, NV, United States) was used for the statistical analyses. All data are presented as the means ± standard error. Statistical analysis was performed using Student’s *t*-test or one-way ANOVA, with *P* < 0.05 considered statistically significant.

## Results

### BRCC3 Was Upregulated in Bladder Cancer

**FIGURE 1:**
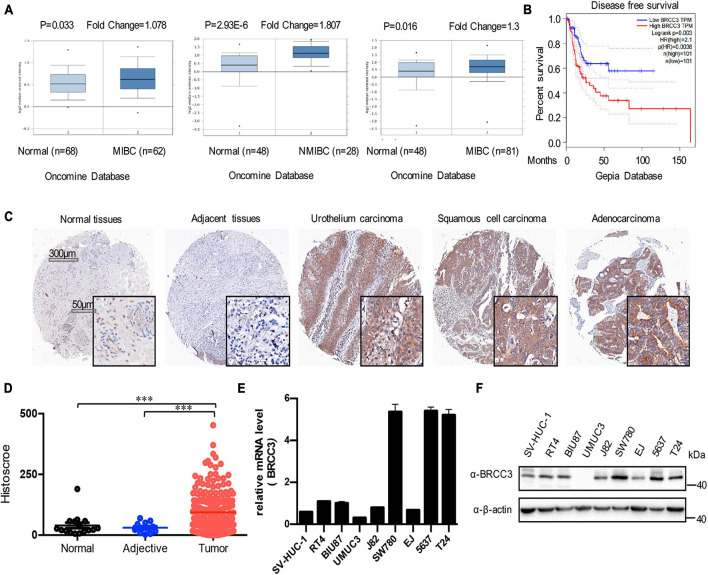
Expression of BRCC3 in bladder cancer. **(A)** Data form Oncomine database. Three microarray datasets exhibited obvious upregulation of BRCC3 expression in muscle-invading bladder cancer tissue compared to normal. **(B)** Disease-free survival time analysis of data from the GEPIA database. The *p*-value is indicated. **(C)** Immunohistochemical staining for BRCC3 in tissue microarrays containing cancer tissue samples, adjacent tissue samples and normal bladder tissue samples from patients with chronic cystitis or a healthy urinary tract. Representative staining images for different pathological types of bladder cancer including uro-endothelial carcinoma, squamous cell carcinomas and adenocarcinoma are shown. **(D)** Tissue microarray data analysis of BRCC3 expression in 188 bladder cancer tissue samples, 12 corresponding adjacent tissue samples and 16 normal bladder tissue samples (including 8 chronic cystitis tissue samples and 8 healthy bladder tissue samples collected at autopsy). **(E)** The mRNA expression levels of BRCC3 in SV-HUC-1 and 8 bladder cancer cell lines. **(F)** The protein expression levels of BRCC3 in SV-HUC-1 and 8 bladder cancer cell lines. *P < 0.05, **P < 0.001, and ***P < 0.001 compared with controls.

To analyze the level of BRCC3 in bladder cancer, we first searched the Oncomine and GEPIA databases. The mRNA levels of BRCC3 in bladder cancer tissue specimens were obviously upregulated, compared with those in normal bladder tissue samples (Figure 1A). And upregulated BRCC3 expression was negatively related to the diseases-free survival in patients with bladder cancer (Figure 1B). To confirm the results above, we performed an IHC analysis of BRCC3 using tissue microarrays, which contained 188 bladder cancer tissue samples, 12 corresponding adjacent tissue samples and 16 normal bladder tissue samples. The results indicated a significant upregulation of the BRCC3 expression level in the cancer tissue compared to the paired adjacent and normal bladder tissue (Figures 1C,D). Moreover, we investigated the BRCC3 mRNA and protein levels in bladder cancer-derived cell lines and the immortalized normal uroepithelial cell line SV-HUC1, and found that 6 out of 8 cancer cell lines showed BRCC3 expression upregulation, compared with SV-HUC1 (Figures 1E,F). Taken together, these results implied BRCC3 was upregulated aberrantly in bladder cancer.

### Overexpression of BRCC3 Increased Cell Proliferation and Migration

**FIGURE 2:**
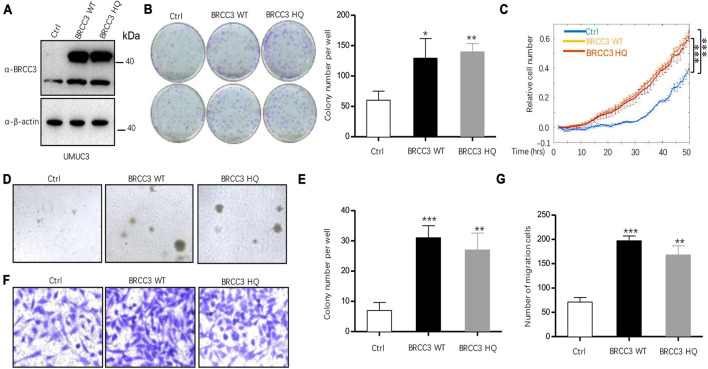
The overexpression of BRCC3 increased tumor cell proliferation *in vitro*. **(A)** BRCC3-overexpressing cells were identified via immunoblotting. **(B)** Colony formation assays. The number of clones was counted and plotted. **(C)** An ECIS cell proliferation assay was performed. **(D)** Three-dimensional colony formation assay was performed in soft agar. The colony numbers are expressed as the means ± standard error of triplicate assays. **(E)** The Quantitative data show the colonies numbers in **(D)**. **(F)** Transwell migration assay. The relative cell numbers of migrating were statistically analyzed. **(G)** Quantitative data show the number of migrating cells in **(F)**. *P< 0.05, **P< 0.001, and ***P< 0.001 compared with controls.

To study the role of BRCC3 in the biological behaviors, we created wild type BRCC3 (BRCC3 WT) overexpression and BRCC3 deubiquitinating enzyme-null mutant (H122Q, BRCC3 HQ) overexpression UMUC3 bladder cancer cell lines via lentiviral transfection. Western blotting determined the protein levels of the wild type and the mutant type BRCC3 (Figure 2A). The colony formation assay and cell growth assay based on the ECIS system showed that the overexpression of the wild type BRCC3 dramatically promoted cell growth and colony formation (Figures 2B,C). Next, we performed soft agar assays which showed a similar result to the results of the colony formation and proliferation assays (Figures 2D,E). Furthermore, a transwell migration assay suggested the overexpression of the wild type BRCC3 increased cell migration (Figures 2F,G). And meanwhile we investigated the effects of the overexpression of the deubiquitinating enzyme-null mutant BRCC3 on both cell proliferation and migration, which interestingly showed the similar effects as the wide type protein (Figures 2B–D,F).

### Knocking Out BRCC3 Inhibited Cell Proliferation and Migration

**FIGURE 3:**
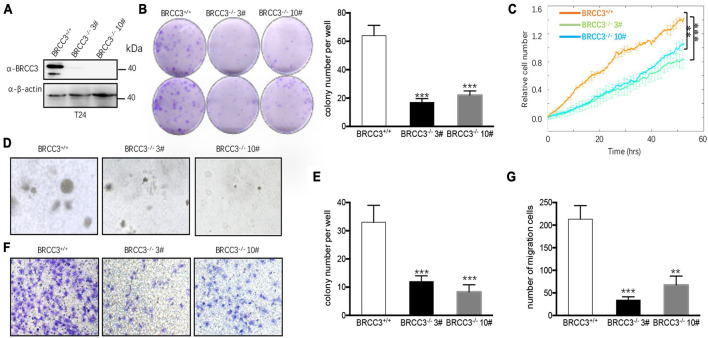
The depletion of BRCC3 repressed tumor formation *in vitro*. **(A)** BRCC3-deficient cells were identified via Western blotting assays. **(B)** Colony formation assays. **(C)** An ECIS cell proliferation assay was performed. **(D)** Three-dimensional colony formation assay was performed in soft agar. The colony numbers are expressed as the means ± standard error of triplicate assays. **(E)** The Quantitative data show the colonies numbers in **(D)**. **(F)** Transwell migration assay. The relative cell numbers of migrating were statistically analyzed. **(G)** Quantitative data show the number of migrating cells in **(F)**. *P< 0.05, **P< 0.001, and ***P< 0.001 compared with controls.

We then knocked out the endogenous BRCC3 gene in the T24 cell line using CRISPR-Cas9 gene-editing system. Western blot analysis verified the abolition of the expression of BRCC3 protein in the knockout cells (Figure 3A). Colony formation assays and the ECIS proliferation system were used to measure cell proliferation. BRCC3-deficient cells showed significant inhibition of cell growth (Figures 3B,C). The colony numbers in soft agar assays were remarkably decreased when BRCC3 expression was depleted (Figures 3D,E). A transwell migration assay suggested that knocking out BRCC3 reduced cell migration obviously (Figures 3F,G).

### Genetic Deficiency in BRCC3 Resulted in Inactivation of the NF-κB Signaling Pathway

**FIGURE 4:**
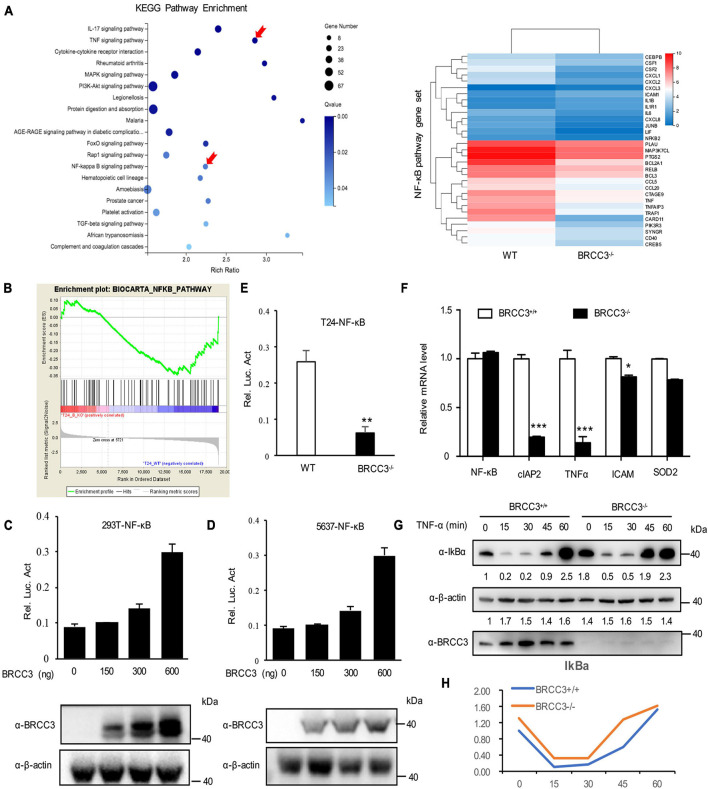
Knocking out of BRCC3 resulted in inactivation of the NF-κB signaling pathway. **(A)** KEGG pathway enrichment analysis was used to assess wild type and BRCC3^–/–^ bladder cancer cells. **(B)** GSEA identified NF-κB pathway-related gene sets enriched in the wild-type cells. **(C)** BRCC3 promotes the transcriptional activity of NF-κB. 293T cells were transfected with an NF-κB reporter firefly luciferase plasmid (200 ng), pRL-TK (10 ng) and the indicated amounts of a BRCC3 plasmid. Reporter assays were performed 48 h after transfection, and the results are presented as the NF-κB/TK luciferase activity. Data were analyzed employing one-way ANOVA and presented as the means ± standard error (*n* = 3/group). **(D)** BRCC3 promoted the transcriptional activity of NF-κB in bladder cancer 5637 bladder cancer cells. The experiments and data analyses were performed as in **(C)**. **(E)** BRCC3 ablation inhibited the transcriptional activity of NF-κB. **(F)** The BRCC3 deficiency blocked the transcription of NF-κB-targeted genes in the T24 cells. The expression levels of the indicated NF-κB-targeted genes were examined by RT-qPCR in BRCC3^–/–^ T24 cells and wild type T24 cells. **(G)** BRCC3 deficiency reduced the degradation of IκBα under TNFα stimulation. BRCC3^–/–^ T24 cells and wild type T24 cells were treated with 5 ng/ml TNFα for indicated time, and then the cell total protein lysis was analyzed by immunoblotting assays. **(H)** Quantitative data show the relative protein levels of IκBα in **(G)**. Statistical analysis was conducted using a *t*-test. The means ± standard error from three independent experiments is shown. *P< 0.05, **P< 0.001, and ***P< 0.001 compared with controls.

To identify the possible signaling pathway involved, we performed RNA-Seq analysis using the wild type and BRCC3-deficient T24 cell lines. Gene Set Enrichment Analysis (GSEA) was conducted using KEGG pathway enrichment models, and we found that the NF-κB signaling pathway was significantly inactivated when BRCC3 was knocked out (Figures 4A,B). To confirm this finding, we tested whether BRCC3 can activate the luciferase activity of an NF-κB reporter in both HEK293 cells and three bladder cancer cell lines 5637, T24, and EJ. We observed that exogenous overexpression of BRCC3 activated NF-κB pathway in a dose-dependent manner (Figures 4C,D and Supplementary Figure 1). To identify the role of endogenous BRCC3, an NF-κB reporter assay was performed with wide type and BRCC3-deficient T24 cells. The results showed that the ablation of BRCC3 vastly inhibited NF-κB activity (Figure 4E). In addition, Real-time PCR analyses indicated that the ablation of BRCC3 reduced the expression of the NF-κB signaling down-stream genes including cIAP2, TNFα, and ICAM (Figure 4F). It is well recognized that IκBα protein sequesters P65 in the cytoplasm under physiological conditions. However, under stimulation with cytokines, pathogens and so on, IκBα is degraded which makes P65 released into the nucleus, leading to NF-κB activation finally. Therefore, we also checked the influence of BRCC3 on the degradation rate of IκBα under TNFα stimulation. We found that abolition of BRCC3 reduced IκB degradation (Figures 4G,H), which lead to the blockade of NF-κB activation.

### BRCC3 Activated NF-κB Signaling Pathway via TRAF2

**FIGURE 5:**
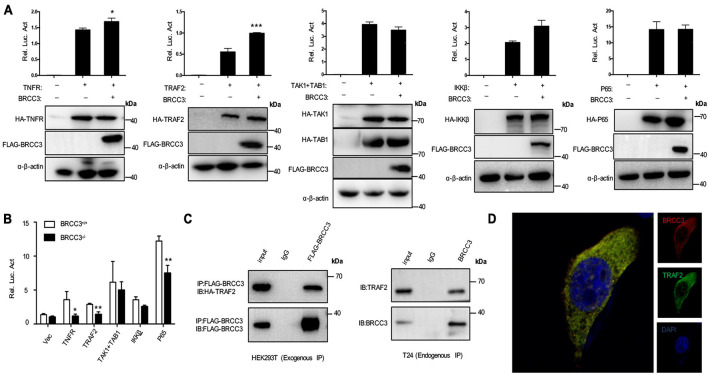
BRCC3 activated the NF-κB signaling pathway through TRAF2. **(A)** The synergistic effects of TNFR, TRAF2, TAK1, and TAB1, IKKβ or P65 together with BRCC3 on NF-κB transcriptional activation were evaluated. HEK293T cells were co-transfected with an NF-κB reporter firefly luciferase plasmid (200 ng), pRL-TK (10 ng), the indicated component of an NF-κB pathway (300 ng) and a BRCC3 plasmid (300 ng). Reporter assays were performed 48 h after transfection, and the results are presented as the NF-κB/TK luciferase activity. Data were analyzed employing one-way ANOVA and are presented as the mean ± SE (*n* = 3 per group). **(B)** The influence of endogenous BRCC3 on NF-κB transcriptional activation induced by different NF-κB pathway components including TNFR, TRAF2, TAK1 and TAB1, IKKβ, and P65 was assessed. **(C)** BRCC3 interacted with TRAF2 in both exogenous and endogenous ways. The whole-cell extracts of HEK293T cells (co-transfected with BRCC3 and TRAF2 for 48 h) or the wild type T24 cells were prepared and incubated with an anti-Flag antibody or anti-BRCC3 antibody and Sepharose beads, and the immunoprecipitants were collected. The precipitates were then analyzed by SDS–PAGE, and Western blotting was performed with an anti-HA antibody or anti-TRAF2 antibody. Data are shown as the means ± standard error of technical replicates from one representative experiment out of three experiments. *P< 0.05, **P< 0.001, ***P< 0.001 compared with controls. **(D)** Immunofluorescence staining showed the co-localization of endogenous BRCC3 and TRAF2. Fluorescence confocal microscopy analysis was performed with T24 cells, using antibodies of BRCC3 and TRAF2 according to the standard immunofluorescence assays.

To further investigate the relationship between BRCC3 and NF-κB signaling, BRCC3 was co-expressed with several NF-κB signaling protein plasmids in HEK293T cells and then analyzed the relative luciferase activity using an NF-κB signaling reporter plasmid. We found that BRCC3 increased the activation NF-κB signaling pathway together with the upstream molecules TNFR and TRAF2 (Figure 5A). However, BRCC3 did not increase IKKβ, P65 and TAK1/TAB1-induced activation of NF-κB signaling (Figure 5A). Besides, we found overexpression of TNFR, TRAF2 and P65 were able to activate the NF-κB signaling pathway in wild type (BRCC3^+/+^) T24 cells, but failed to activate the signaling in BRCC3-deficient (BRCC3^–/–^) T24 cells (Figure 5B). These results suggested the activation of the NF-κB signaling pathway was at least partially dependent on the function of BRCC3. Additionally, we used immunoprecipitation assays to examine the interaction of BRCC3 with proteins of components of NF-κB signaling pathways such as TNFR, TRAF2, P65, and so on. We found that BRCC3 interacted with TRAF2 in both exogenous and endogenous expression models (Figure 5C). Moreover, the colocalization of BRCC3 and TRAF2 was proved by an endogenous immunofluorescence (Figure 5D).

### Knocking Out BRCC3 Inhibited Bladder Cancer Growth *in vivo*

**FIGURE 6:**
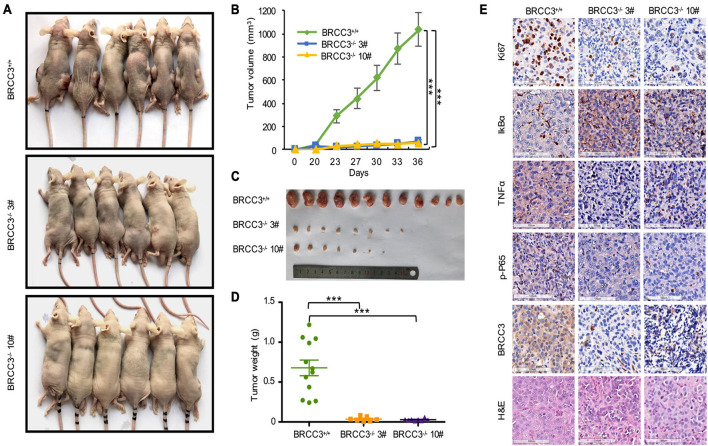
The depletion of BRCC3 inhibits bladder cancer tumorigenesis *in vivo*. **(A)** Xenograft models (*n* = 6) were established by subcutaneously inoculating wild-type or BRCC3-KO cells and allowing the cells to grow for 5 weeks. Then the mice were sacrificed and the tumors were removed and weighed. **(B–D)** The tumor volume and weight measurements are shown. **(E)** Representative H&E staining and immunohistochemical staining of the xenograft tumors from the tumor-bearing mice in the wild-type and BRCC3-KO groups, showed the deficiency of BRCC3 (the fifth panel) and the upregulation of IκBα expression (the second panel), the downregulation of Ki67 expression (the top panel), TNFα expression (the third panel) and p-P65 expression (the fourth panel) in the BRCC3-KO groups. Statistical analysis of the tumor volume and weight were performed using one-way ANOVA. The means ± standard error of three independent experiments is shown. *P < 0.05, **P < 0.001, and ***P < 0.001 compared with controls.

To confirm BRCC3 function *in vivo*, we constructed xenograft models. The parental T24 cells and two BRCC3-deficient T24 cell lines were injected into separate nude mice. About 1 month later, the tumors were dissected from the tumor-bearing mice. The volumes of the tumors from the parental T24 cells were much larger than those from the BRCC3^–/–^ cells (Figures 6A,B). Accordingly, a dramatic advantage in tumor weight that agreed with the differences in tumor size was also observed (Figures 6C,D). These results suggested that BRCC3 played an important role in the regulation of bladder cancer tumorigenesis *in vivo*. Moreover, the dissected neoplasms were embedded in paraffin and assessed by immunohistochemistry, finding that the expression of Ki67 was lower in the BRCC3^–/–^ group (Figure 6E). Furthermore, TNFα and phospho-P65 was decreased while the level of IκBα was increased in the BRCC3^–/–^ group, which confirmed that the abolition of BRCC3 inhibited the NF-κB signaling pathway (Figure 6E).

## Discussion

The deubiquitination family is involved in a wide range of biological processes, including cancer and inflammation (Massoumi, 2011; Li et al., 2013). The function of BRCC3 in bladder cancer remains elusive. The protein level of BRCC3 in bladder cancer in The Cancer Genome Atlas (TCGA) datasets showed that BRCC3 expression is aberrantly upregulated in bladder cancer patients. Analysis of Oncomine datasets was consistent with this conclusion. To further verify these findings, IHC staining found upregulated BRCC3 expression in bladder cancer tumor tissue. Next, we determined the function ofBRCC3 in the progression of bladder cancer using BRCC3 over-expressed and BRCC3 deficient cells *in vitro*. Our findings showed BRCC3 plays a crucial role in facilitating the development and progression of bladder cancer.

To reveal the way how BRCC3 promotes carcinogenesis, we analyzed BRCC3-deficient bladder cancer cell lines by RNA-Seq, and the data showed that the NF-κB inflammatory pathway was notably downregulated when BRCC3 was knockout. We further confirmed this result by luciferase, qPCR and Western blotting assays. Through co-transfecting of the node protein of the NF-κB pathway, we reported that BRCC3 maximizes the potential of TRAF2 to activate the NF-κB pathway, and further exploration showed that BRCC3 binds to TRAF2. Finally, the xenograft model showed that a deficiency in BRCC3 expression significantly decreased tumorigenesis *in vivo*.

Accumulating evidence indicates that the deubiquitinase BRCC3 participates in carcinogenesis by regulating DNA stability (Huang et al., 2015). Although BRCC3 has been reported to be an important regulator of NLRP3 activity (Py et al., 2013; Hu et al., 2019), the function of BRCC3 in the NF-κB signaling pathway has not been reported. Our results showed BRCC3 promotes the NF-κB signaling in bladder cancer, therefore resulting in tumorigenesis, which is different from the previously described mechanism. TRAF2 can promote tumorigenesis in several cancers (Wu et al., 2005; Etemadi et al., 2015; Borghi et al., 2016; Wei et al., 2017). The stability and activity of TRAF2 are mediated by a large number of E3 ubiquitin ligases and deubiquitinating enzymes (Habelhah et al., 2002; Trompouki et al., 2003; Li et al., 2009; Xiao et al., 2012; Zhong et al., 2013; Borghi et al., 2016). We demonstrated that BRCC3 facilitates tumorigenesis via TRAF2 in bladder cancer. Interestingly, by using a validated deubiquitinating enzyme-null site-directed mutation (H122Q) of BRCC3 (Cooper et al., 2009; Py et al., 2013; Liu et al., 2018), we found that BRCC3 interacts with TRAF2, yet through a mechanism that is independent of the deubiquitinating enzyme activity of BRCC3.

**FIGURE 7:**
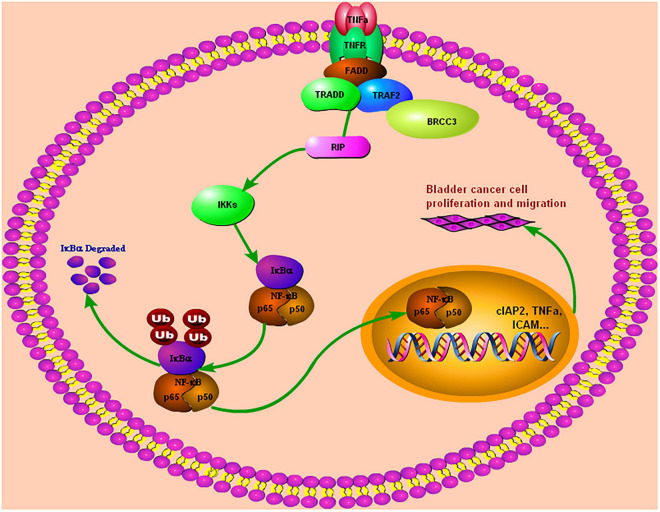
Our study demonstrated that BRCC3 could promote BCa cells proliferation and migration both *in vitro* and *in vivo*. Furthermore, BRCC3 exerts its oncogenic role via binding to TRAF2, which in turn activates NF-κB signaling and inflammation in bladder cancer.

In summary, we found that BRCC3 is overexpressed and associated with a poor prognosis in bladder cancer. BRCC3 exerts its oncogenic role via binding to TRAF2, which in turn leads the activation of NF-κB signaling in bladder cancer (Figure 7). Our study provides novel insight into the function of BRCC3 in the TRAF2-activating NF-κB signaling cascade in bladder cancer.

## Data Availability Statement

Oncomine data was downloaded from Thermo Fisher Scientific institute (https://www.oncomine.org/resource/login.html). Gene Expression Profiling Interactive Analysis (GEPIA) data was downloaded from Zhang’s Lab of Perking university (http://gepia.cancer-pku.cn/).

## Ethics Statement

The studies involving human participants were reviewed and approved by the Ethics Committee at Zhongnan Hospital of Wuhan University. Written informed consent for participation was not required for this study in accordance with the national legislation and the institutional requirements. The animal study was reviewed and approved by the Ethics Committee at Zhongnan Hospital of Wuhan University.

## Author Contributions

HT, YY, YL, and ZH performed the majority of the experiments and prepared the manuscript. JZ performed Luciferase reporter assays and IHC experiments. YL and TL wrote the main manuscript. TL, SL, JP, and XW designed the research. All authors read and approved the manuscript.

## Conflict of Interest

The authors declare that the research was conducted in the absence of any commercial or financial relationships that could be construed as a potential conflict of interest.

## Publisher’s Note

All claims expressed in this article are solely those of the authors and do not necessarily represent those of their affiliated organizations, or those of the publisher, the editors and the reviewers. Any product that may be evaluated in this article, or claim that may be made by its manufacturer, is not guaranteed or endorsed by the publisher.

## Footnotes

1
http://www.e-crisp.org/E-CRISP/

## Supplementary Material

Supplementary Figure 1 BRCC3 promotes the transcriptional activity of NF-κB. T24 or EJ cells were transfected with an NF-κB reporter firefly luciferase plasmid (200 ng), pRL-TK (10 ng) and the indicated amounts of a BRCC3 plasmid. Reporter assays were performed 48 h after transfection, and the results are presented as the NF-κB/TK luciferase activity. Data were analyzed employing one-way ANOVA and presented as the means ± standard error (*n* = 3/group).

## Abbreviations

BCa: Bladder cancer
CHX: cycloheximide
DMEM: Dulbecco’s modified Eagle’s medium
DSBs: DNA double-strand breaks
ECIS: Electric Cell-substrate Impedance Sensing
EMT: epithelial-mesenchymal transition
FBS: fetal bovine serum
IHC: Immunohistochemical
K63-Ub: lysine 63-linked ubiquitin polymers
NF-κB: Nuclear factor kappa-B
PBS: phosphate-buffered saline
qRT-PCR: quantitative real-time PCR
RT: Reverse transcription
SDS-PAGE: sodium dodecyl sulfate-polyacrylamide gel electrophoresis
sgRNAs: small guide RNAs.
