# Supplementary figures and images for: BRCC3 Promotes Tumorigenesis of Bladder Cancer by Activating the NF-κB Signaling Pathway Through Targeting TRAF2
Source: Front Cell Dev Biol. 2021 Sep 16;9:720349. doi: 10.3389/fcell.2021.720349 (PMC8481630; doi:10.3389/fcell.2021.720349)

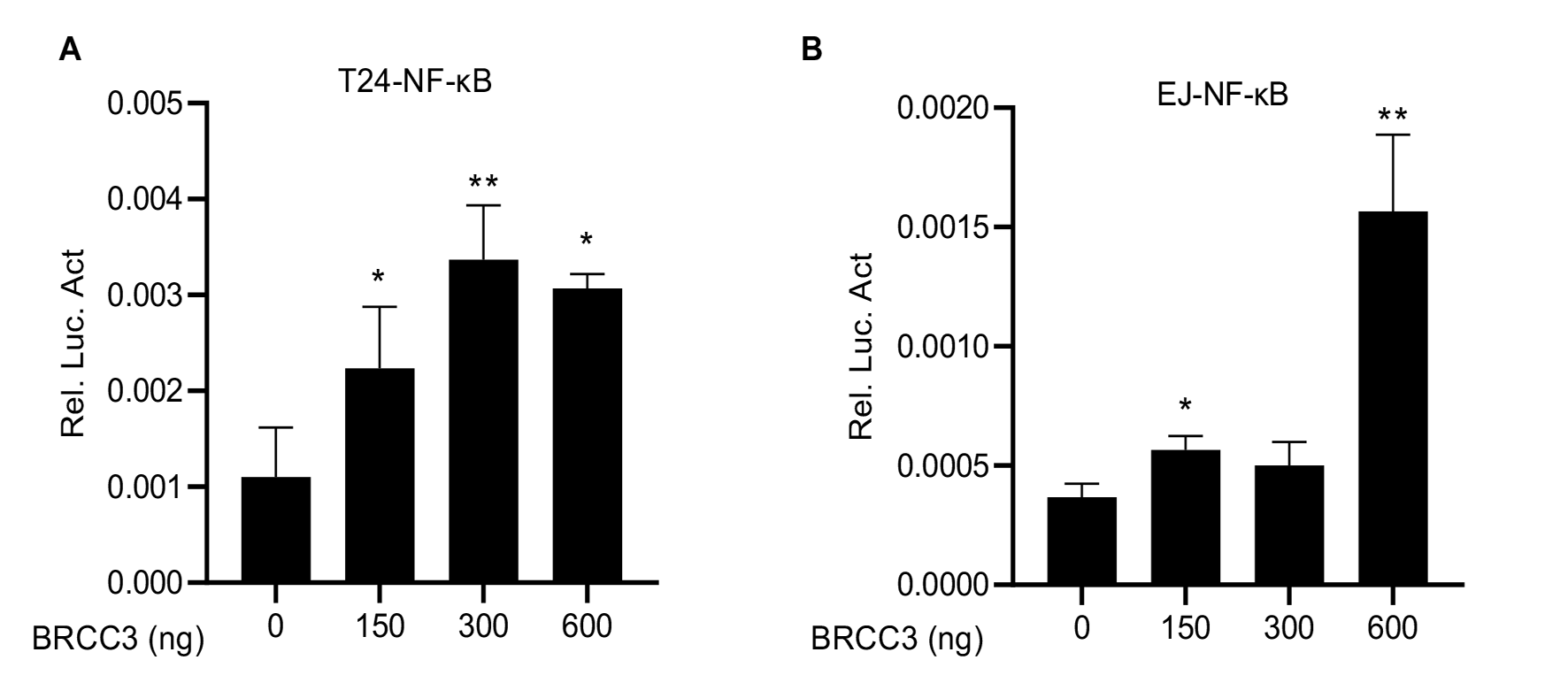

Supplement: Supplementary Figure 1 — BRCC3 promotes the transcriptional activity of NF-κB. T24 or EJ cells were transfected with an NF-κB reporter firefly luciferase plasmid (200 ng), pRL-TK (10 ng) and the indicated amounts of a BRCC3 plasmid. Reporter assays were performed 48 h after transfection, and the results are presented as the NF-κB/TK luciferase activity. Data were analyzed employing one-way ANOVA and presented as the means ± standard error (n = 3/group). [file Image_1.TIF]
