# Supplementary material for: BRCC3 Promotes Tumorigenesis of Bladder Cancer by Activating the NF-κB Signaling Pathway Through Targeting TRAF2
Source: Front Cell Dev Biol. 2021 Sep 16;9:720349. doi: 10.3389/fcell.2021.720349 (PMC8481630; doi:10.3389/fcell.2021.720349)
Supplement: Supplementary file 2 [file Table_1.docx]

**BRCC3 Promotes Tumourigenesis of Bladder Cancer by Activating** **the NF-κB Signal Pathway through Targeting TRAF2**

Huangheng Tao^1,2,3‡^, Yixiang Liao^4,5‡^, Youji Yan^4,5‡^, Zhiwen He^1,2‡^, Jiajie Zhou^4,5^,

Xinghuan Wang^1,2†^, Jianping Peng^1,2†^, Shangze Li^6,7†^ , Tao Liu^1,2†^

**Supplementary Information**

**Supplementary Table S1. List of primers for qRT-PCR.**

| **Gene name** | **Forward primer (5’-3’)** | **Reverse primer (5’-3’)** | **Length (bp)** |
| --- | --- | --- | --- |
| cIAP2 | TCAAGTTCAAGCCAGTTACC | GACTCTGCATTTTCATCTCC | 81 |
| ICAM1 | TCAGTGTGACCGCAGAG GACGA | TTGGGCGCCGGAAAGCTGTAGAT | 115 |
| TNFα | GCCGCATCGCCGTCTCCTAC | CCTCAGCCCCCTCTGG GGTC | 90 |
| NFKB1 | TGGTATCAGACGCCAT CTA | GCTGTCCTGTCCATTCTTAC | 76 |
| SOD2 | GCACGCTTACTACCTTCAGT | CTCCCAGTTGATTACATTCC | 76 |
| GAPDH | ACAACTTTGGTATCGTGGAAGG | GCCATCACGCCACAGTTTC | 101 |
